# Parents’ Knowledge of the Impact of Cystic Fibrosis on the Quality of Life of Children and Adolescents Suffering from This Disease as an Element of Patient Safety

**DOI:** 10.3390/jcm12165214

**Published:** 2023-08-10

**Authors:** Grażyna Dykowska, Ewa Śmigrocka, Urszula Borawska-Kowalczyk, Dorota Sands, Zofia Sienkiewicz, Anna Leńczuk-Gruba, Damian Gorczyca, Mariola Głowacka

**Affiliations:** 1 Department of Health Economics and Medical Law, Faculty of Health Sciences, Medical University of Warsaw, 02-091 Warsaw, Poland; 2 Cystic Fibrosis Department, Institute of Mother and Child, 01-211 Warsaw, Poland; ewa.24@vp.pl (E.Ś.); ula.borawska@wp.pl (U.B.-K.); dorota.sands@imid.med.pl (D.S.); 3 Cystic Fibrosis Center, Children’s Hospital in Dziekanów Leśny, 05-092 Warsaw, Poland; 4 Department of Nursing, Social and Medical Development, Medical University of Warsaw, 02-091 Warsaw, Poland; zofia.sienkiewicz@wum.edu.pl (Z.S.); anna.lenczuk.gruba@wum.edu.pl (A.L.-G.); 5 Medical Faculty, Lazarski University, 02-662 Warsaw, Poland; damian.gorczyca@lazarski.pl; 6 The Mazovian Academy in Płock, 08-110 Płock, Poland; m.glowacka@mazowiecka.edu.pl

**Keywords:** cystic fibrosis (CF), child, quality of life, parents, safety

## Abstract

Parental perspective on the health, safety, and quality of life in children and adolescents with cystic fibrosis (CF). Aim of the study: Assessment of the impact of a chronic disease such as cystic fibrosis (CF) on the quality of life and safety of children and adolescents as perceived by parents/caretakers. Methods: The study was conducted at the Department of Lung Diseases of the Institute of Mother and Child, a branch of the Cystic Fibrosis Centre Children of Warsaw SZPZOZ in Dziekanów Leśny, the largest pediatric CF center in Poland, and in the Rodzinamuko group on Facebook. A total of 139 parents participated in the study. The study was conducted using the diagnostic survey method with the use of the Kid- & Kiddo-KINDLR questionnaire for examining the quality of life of children and adolescents and a demographic questionnaire. Results: The perception of cystic fibrosis (CF) as a chronic disease varies based on parental residence and professional status. The well-being of children and adolescents with CF is tied to their parents’ employment, particularly regarding schooling. Social interactions are influenced by the level of parental education. The quality of life in children and adolescents with CF is age-dependent, with younger children exhibiting higher quality of life. This age–quality of life relationship extends to physical well-being, emotional well-being, and school-related aspects. Furthermore, the emotional dimension of quality of life is affected by the child’s age at the time of diagnosis. Conclusions: The Kid- & Kiddo-KINDLR QoL Questionnaire for children with cystic fibrosis is a good tool to measure parental knowledge. The study shows the need for the whole family to understand and be aware of the impact of CF on family life. Parents may be tired or may misunderstand or miscommunicate the medical team’s instructions, which may affect both family life and patient safety. To ensure patient safety, parents should work with healthcare professionals at hospitals or clinics but also at home. They should also account for the family as a whole, not just for the problems of the child with CF.

## 1. Introduction

Each chronic disease adversely affects the functioning of children and adolescents, being a long-term burdening factor that affects their physical, mental, and social development [1]. Patient self-assessment of health-related quality of life is an important determinant of a properly running therapeutic and nursing process. Improvement in clinical parameters and general indicators does not always translate to better well-being of the patient and does not always contribute to better self-perception of health [2]. In the case of chronically ill people, there are processes that make their subjective assessment of their health very different from the objective status. On the one hand, there is a change in self-perception from a healthy to a sick person, and on the other, the patient adapts to the new situation and limitations related to the disease and becomes accustomed to “being sick” [2].

The assessment of quality of life makes it possible to adjust the treatment to the patient’s needs, because the aim of treatment is not only to prolong the patient’s life but above all to improve his/her well-being and overall quality of life [3].

Cystic fibrosis (CF) is a multi-organ disease with a varied course and clinical symptoms. According to the ECFS Patient Registry Annual Report 2020, there were 1341 CF patients in Poland in 2019, of which 66.74% were children under the age of 18 (889 patients) and 33.26% were adults (443). The median age of the patients was 13.2 years. The gender distribution was even: women—49.9%, men—50.1% [3]. CF is a high-maintenance disease that requires both the parents and the child to make everyday life compatible with the treatment and to take the necessary steps to prevent exacerbations of the disease and subsequent hospitalizations. Limitations occur that affect the patient’s family and social life. A patient with CF should be subject to comprehensive medical care provided by a properly organized multidisciplinary team. According to Conway et al., this significantly affects patient safety, and such a treatment team should comprise consultants, a clinical nurse, a microbiologist, a physiotherapist, a dietician, a pharmacist, a clinical psychologist, a social worker, a clinical geneticist, and other healthcare professionals, all of whom should be experienced in the care of patients with CF [4].

An improvement in physical indicators does not always translate to better well-being of CF patients and better self-perception of health. A child’s self-esteem is also affected by insufficient public knowledge of the disease, which may result in prejudice against and social exclusion of CF patients. Stigma, isolation, and social exclusion contribute to lower self-assessed health-related quality of life [5].

### 1.1. Aim of the Study

The aim of the study is to complete an assessment of the impact of a chronic disease such as CF on the quality of life and safety of children and adolescents as perceived by parents/caretakers.

### 1.2. Materials and Methods

The study used a diagnostic survey method with a standardized questionnaire, Kid- & Kiddo-KINDL^R^, and a research group questionnaire. The standardized Kid- & Kiddo-KINDL^R^ questionnaire was used to assess the quality of life of children and adolescents (version intended for parents of children aged 7–17). The questionnaire was developed by a team led by Professor Ulrike Ravens-Sieberer, PhD in philosophy, from the University Medical Centre Hamburg-Eppendorf, and its use was approved by its authors [6,7]. The Kid- & Kiddo-KINDL^R^ questionnaire is a generic instrument for recording the health-related quality of life of children and adolescents. It is used to record the quality of life of both healthy and sick children. There are three versions of the questionnaire for different age groups and developmental stages. Surveys for children and surveys for caregivers are offered for the version for each age [8]. The basic module consists of 24 questions in 6 areas related to physical well-being, emotional well-being, self-esteem, family, social interactions, and the school environment. In the questionnaire, only one area refers to physical well-being, whereas mental health is subdivided into emotional well-being and self-esteem. The social functioning of children and adolescents comprises areas related to family life, social interactions, and school. The basic version of the questionnaire is supplemented with six questions from an additional module on the quality of life related to a chronic disease or longer hospitalization. Respondents answer the questions on a 5-point Likert scale, where 1 = never, 2 = rarely, 3 = sometimes, 4 = often, and 5 = all the time. It is also possible to transform all scales so that the values range from 0 to 100, with higher values indicating a better quality of life [9]. For questions 1, 2, and 3 about physical well-being; questions 2, 3, and 4 about emotional well-being; questions 3 and 4 about family life; question 4 about social contacts; and question 4 about school, 1 = 5, 2 = 4, 3 = 3, 4 = 2, and 5 = 1, where answer 5 (all the time) is the positive end of the position. This also applies to questions 1, 2, 4, and 5 and question 6 about the illness [10]. The questionnaire measures the child’s experiences from the preceding week. It also includes questions about the child’s gender and age. The Polish version of the questionnaire is available at: www.kindl.org/deutsch accessed on 17 May 2021 [11].

The next section of the questionnaire consists of an additional 13 closed questions (single choice) concerning the diagnosis, daily respiratory rehabilitation, and the frequency of CF symptoms, as well as socio-demographic questions on gender, age, marital status, place of residence, education, and professional situation.

The study was conducted at the Department of Lung Diseases of the Institute of Mother and Child, a branch of the Cystic Fibrosis Centre Children of Warsaw SZPZOZ in Dziekanów Leśny, the largest pediatric CF center in Poland, from 6 July to 1 December 2021. The study was conducted with the approval of the Head of the Centre and Hospital Management and the hospital’s lawyer, as well as the odrinator of the cystic fibrosis ward (professor pediatrician). They provided their consent to study No. 968/2021 and involved the parents of children hospitalized in the above-mentioned department. Part of the results were obtained via the Rodzinamuko private group on Facebook. The study was conducted in accordance with the Declaration of Helsinki and did not involve medical research on humans, including research on biological material from an identified recipient and research on medical data from patients’ medical records. It received consent from the Hospital Management for the survey of parents.

## 2. Methods

Data analysis was performed using SPSS Statistics version 23.0 and an Excel spreadsheet. In order to verify significant differences between two independent groups, the Mann–Whitney U rank test was used. Spearman’s rho correlation coefficient was used to examine the relationship between the variables. The chi^2^ test of independence was used to examine the correlations between variables on nominal scales. Statistical inference was performed at the standardized significance level of *p* < 0.05.

## 3. Results

Health-related quality of life of children and adolescents—analysis of questions from the Kid- & Kiddo-KINDL^R^ questionnaire.

### 3.1. Quality of Life Regarding Physical Well-Being

**During the past week my child felt ill**: 42.4% of the respondents stated that their child “never” felt sick during the previous week and 31.7% of parents answered “seldom”. “Sometimes” was indicated by 19.5% of parents, “often” by 4.35% of parents, and “all the time” by 2.1% of parents.

**During the past week my child had a headache or tummyache**: 51% of the respondents stated that the child “never” experienced headaches and abdominal pain, and 30.9% of the respondents stated that these complaints occurred “seldom”. Others indicated “sometimes”. None of the respondents answered that these pains occurred “all the time”.

**During the past week my child was tired and worn out:** 40.3% of the respondents stated that the child was “seldom” tired or worn out. The child felt tired “sometimes” according to 25.9% of the respondents. Other respondents indicated that the child was “often” tired and worn out.

**During the past week my child felt strong and full of energy:** 38.1% of the respondents stated that this would “often” be true. The child was “sometimes” full of energy according to 26.6% of the respondents. “All the time” was indicated by 20.9% of respondents and “seldom” or “never” by 14.4% (including “seldom” by 13%).

### 3.2. Quality of Life Regarding Mental Health

#### 3.2.1. Emotional Well-Being

**During the past week my child had fun and laughed a lot**: 51.1% of the respondents stated that the child “often” had fun and laughed a lot; 23.7% and 22.3% of the respondents said their child had fun and laughed a lot “sometimes” and “all the time”, respectively. Other respondents indicated that the child “seldom” had fun and laughed a lot.

**During the past week my child didn’t feel much like doing anything**: 42.4% of parents stated that the child was “seldom” bored. According to 36% of parents the child was “often” bored. The child was “never” bored according to 14.4% of the respondents. Other respondents indicated that the child was bored “sometimes”.

**During the past week my child felt alone:** 40.3% of parents stated that their child was “never” lonely, and 38.9% of respondents stated that their child was “seldom” lonely. “Sometimes” was marked by 15.1% of the respondents and “often” by 5.7% of the parents.

**During the past week my child felt scared or unsure of him-/herself:** 52.5% of the respondents stated that their child was “never” afraid or anxious, 26.7% noted such anxiety “rarely”, “sometimes” was indicated by 15.8% of the respondents, and “often” was indicated by 5% of the respondents.

#### 3.2.2. Self-Esteem

**During the past week my child was proud of him-/herself:** 53.3% of the respondents stated that their child was “often” proud of him-/herself, 31.7% said that the child was proud of him-/herself “sometimes”, 10% of the respondents indicated that their child was proud of him-/herself “all the time”, and 1.4% of the respondents stated that the child was “never” proud of him-/herself.

**During the past week my child felt on top of the world:** 47.6% of the respondents stated that their child “often” felt great, 28.8% of the respondents reported that their child felt great “sometimes”, and 12.2% of the parents indicated the child feeling great “all the time”. “Seldom” and “never” were marked by 10% and 1.4% of the respondents, respectively.

**During the past week my child felt pleased with him-/herself:** 55.4% of the respondents stated that their child was “often” pleased with him-/herself, 26.7% reported that their child was pleased with him-/herself only “sometimes”, and 11.5% of the respondents stated that the child was pleased “all the time”. “Seldom” and “never” were marked by 5% and 1.4% of the respondents, respectively.

**During the past week my child had lots of good ideas:** 40.3% of the respondents stated that their child “often” had many good ideas. “Sometimes” and “all the time” were indicated by 27.3% and 26.7% of the respondents, respectively. “Rarely” and “never” were indicated by 5% and 0.7% of the respondents, respectively.

### 3.3. Quality of Life Concerning Social Life

#### 3.3.1. Family

**During the past week my child got on well with us as parents:** 49% of the respondents stated that their child “often” got along well with the parents, 34.5% of the respondents believed that the child had a good relationship with the parents “all the time”, 15.1% of the respondents indicated getting along well with the child “sometimes”, and 1.4% indicated “seldom”.

**During the past week my child felt fine at home:** 58.3% of the respondents stated that their child felt good at home “all the time”, 39.6% indicated this to happen “often”, and 2.1% of the respondents indicated this to be true “sometimes”.

**During the past week we quarreled at home:** 56.8% of the respondents declared that they “seldom” argued at home, 25.2% of the respondents stated that they argued “sometimes”, 3.6% of the parents stated that they argued “often”, and 14.4% of respondents “never” argued.

**During the past week my child has felt that I was bossing him/her around:** 39.6% of the parents declared that the child “seldom” felt bossed around, 28% of the respondents reported that the child “sometimes” felt this way, 22.3% of the respondents indicated that the child felt bossed around by the parent. “Often” and “all the time” were marked by 8% and 2.1% of the respondents, respectively.

#### 3.3.2. Social Contacts

**During the past week my child did things together with friends**. According to 41.7% of the parents, their child “often” did various things together with his/her friends. In the opinion of 21.6% of the respondents this would happen “sometimes”. In 18% of cases this happened “rarely”, and for 7.2% of the respondents this “never” happened. “All the time” was marked by 11.5% and “never” by 7.2% of the respondents.

**During the past week my child was liked by other kids:** 45.3% of the parents stated that the child was “often” liked by other children. Their children were liked “all the time” according to 41.7% of the parents. “Sometimes” was marked by 9.4% of the respondents, “seldom” by 2.9% of the respondents, and “never” by 0.7% of the respondents.

**During the past week my child got along well with his/her friends:** 48.2% of the respondents stated that their child “often” got along well with their friends, 39.6% of the parents indicated that good relations were maintained “all the time”, 7.9% of the respondents stated that their child only “sometimes” get along well with friends, 3.6% of the respondents marked “seldom”, and 0.7% indicated “never” for how often their child got along with their friends.

**During the past week my child felt different from other children:** 38.9% of the parents declared that their child “never” felt that they were different from other children, 27.3% of the respondents believed that they “rarely” felt different, and 4.3% of the respondents stated that the child felt different from other children “all the time”.

#### 3.3.3. School

**During the last week in which my child was at school**, the child easily coped with schoolwork according to 44.6% of the parents. The tasks were easy “all the time” according to 31.7% of the respondents, whereas 18% of the respondents stated that the tasks were easy only “sometimes”. For 5% of the parents the child “seldom” easily coped with schoolwork, and for 0.7% the respondents this was “never” so.

**During the last week in which my child was at school, the child enjoyed the school lessons** according to 39.6% of the parents, and 30.2% of the respondents stated that the child “sometimes” enjoyed the lessons. Only 2.9% of the respondents reported that the child “never” enjoyed the school lessons.

**During the last week in which my child was at school, the child worried about his/her future** according to two groups of respondents, 31.7% each, who indicated “never” and “seldom” as the answer. A total of 10% of the respondents indicated that the child “often” worried about the future, and 2.9% of the respondents indicated that the child was worried about his/her school future “all the time”.

**During the last week in which my child was at school, the child was afraid of bad marks or grades:** 33.8% of the respondents stated that the child was “seldom” afraid of marks or grades, 30.2% of the parents stated that the child was “never” afraid of getting bad marks or grades, 25.9% marked this fear occurring “sometimes”, 9.4% of the respondents reported it occurring “often”, and only in 9.7% of cases did it occur “all the time”.

#### 3.3.4. Quality of Life in Connection with a Chronic Disease or Longer Hospitalization

**During the past week my child was afraid that the illness might get worse**: 30.9% of the respondents felt this way, and 27.3% of the respondents stated that the child “never” had such worries. Occasional fear (“sometimes”) was reported by 20.9% of the respondents and frequent fear (“often”) by 18% of the respondents. A total of 2.9% of respondents stated that their child was constantly worried that their health might deteriorate (“all the time”).

**During the past week my child was sad because of the illness**: 36% of the parents felt this way. In the case of 24.4% of the respondents, the child “seldom” felt sad. Two similarly numerous groups marked two extreme answers, 18% and 18.7%—with the former indicating that the child “never” feel sad and the latter that the child was “often” sad because of their illness. Nearly 2.9% of pointed to their child being sad “all the time”.

**During the past week my child was able to cope well with his/her illness**: 48.9% of the respondents declared that their child “often” coped well with the disease, 24.5% of the parents considered this to happen “sometimes”, and 22.3% of the parents thought it to be true “all the time”. Approximately 4% of the parents (4.3%) assessed it as happening “seldom”.

**During the past week we treated our child as though he/she were younger because of the illness:** 31% of the parents did not treat (“never”) their child as if he/she were younger, and 29.5% of the respondents reported doing so “sometimes”. A total of 23.7% of the respondents declared that they “seldom” treated this way, 10.8% of the parents reported doing so “often”, and 5% of the parents admitted to this happening “all the time”.

**During the last week, my child avoided others noticing his/her illness:** 31.6% of the respondents declared that their child “seldom” did this, and 26.6% of the respondents reported this happening “sometimes”. The respondents claiming that this would “never” happen constituted 20.1% of the study group. For 13.7% of the study participants, this situation would occur “often”.

**During the last week, my child missed something at school because of his/her illness.** This was “often” true to 36% of the respondents, whereas 31% of the parents declared the situation to happen “sometimes”. The parents of 14.4% of the children stated that they would “seldom” miss anything, and 9.3% of cases declared that the child would “never” miss anything. The remaining respondents reported that the child would constantly (“all the time”) miss something happening at school.

### 3.4. Child’s Health Situation—Analysis of Socio-Demographic Questions

A total of 63.3% of the respondents stated that CF was diagnosed in their child in the neonatal period by the age of 28 days. In infancy (by the age of 1), the disease was diagnosed in 23.7% of children. In nearly 10% of the study participants (9.4%), CF was diagnosed in early childhood (the child aged 2 to 5 years). In turn, 3.6% of the respondents declared that their children was diagnosed with the disease at the age of 6 (2.9%) or later, over the age of 10 (0.7%). As many as 80.6% of the children were in a screening program that, among others, can detect CF.

#### Analysis of Socio-Demographics

The study involved 139 participants—one of the parents of a child suffering from CF, mostly women, 83% (*n* = 24). As for the place of residence, 32% (*n* = 45) of the respondents lived in rural areas, 29% of the respondents (*n* = 40) lived in towns with up to 50,000 inhabitants, 14% (*n* = 19) of the respondents lived in cities with a population of 50,000 to 150,000 inhabitants, 10% (*n* = 14) of the respondents lived in cities with a population from 150,000 to 500,000 inhabitants, and 15% (*n* = 21) of the respondents lived in cities with more than 500,000 inhabitants.

As for parental education, 46% of the respondents had secondary education (including 1% of people with lower secondary education), 39% of the respondents had higher education, and 15% of the respondents had vocational education or a lower level of education.

The vast majority of the respondents were married (82%). Every 10th respondent was widowed, divorced, or legally separated. Unmarried respondents accounted for 6% of the study group.

As regards professional status, 55% of the respondents had resigned from professional work due to childcare, 34% of the respondents worked full-time, 5% of the respondents were self-employed, 4% of the respondents were unemployed, 2% of the respondents were students, and 1% of the respondents worked for hire. As regards the gender of the child, 56% of the respondents marked it as female (*n* = 78) and 44% as male (*n* = 61). Approximately one-third (31%) of the children were aged 7–10 years, 31% of the children were aged 11–13 years, and 38% of the children were aged 14–17 years. All the respondents were parents of children suffering from CF.

## 4. Results

The average score for the quality of life of children with CF was 78.17 points (SD = 10.26).

**Figure 1:**
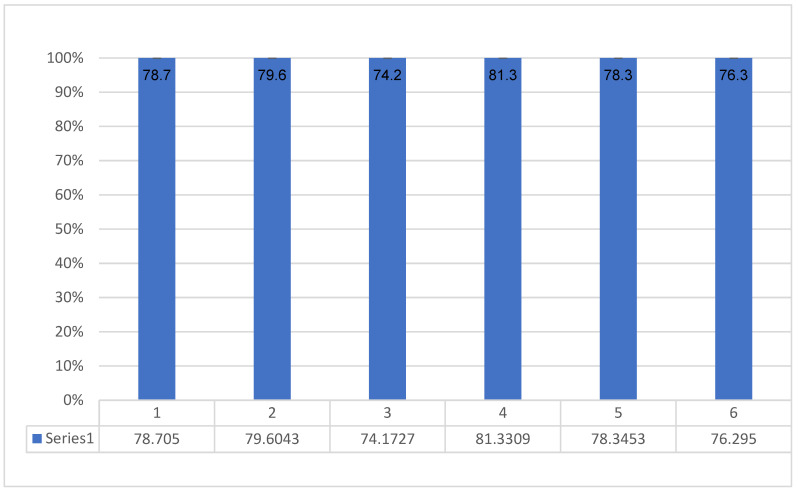
Average scores on the subscales of the KINDL questionnaire.

1. For children’s physical well-being, the average score was 78.7 points (SD = 14.7).
2. A slightly higher score was recorded for emotional well-being, with 79.6 points (SD = 13.2).
3. As regards self-esteem, the average score was the lowest, with 74.2 points (SD = 14.5).
4. On the family subscale, the average number of points was 81.3 (SD = 10.7), and this is the scale where the highest score was recorded.
5. As for social contacts, the average score was 78.3 points (SD = 14.8).
6. On the school scale, the average score was 76.3 points (SD = 14.4) (Figure 1).

A total of 84% (*n* = 117) of respondents reported that their child was hospitalized or had a long-term illness at the time of the survey. Females (*n* = 98) were slightly more likely than males (*n* = 19) to report that their child was currently hospitalized or had a chronic disease (85.2% and 79.2%, respectively). Recognition of CF as a chronic disease did not depend significantly on the sex of the parent (χ^2^_(1)_ = 0.546; *p* > 0.05).

Overall, 75.9% of parents with tertiary education reported that their child was hospitalized with a chronic disease at the time of the study. Among parents with secondary education or less, it was 90%. Parents’ knowledge regarding the qualification of CF as a chronic disease did not depend significantly on their education (χ^2^_(2)_ = 4.532; *p* > 0.05).

A total of 88.2% of the parents who had resigned from work to care for their child reported that their child was hospitalized or had a chronic disease. Similar answers were given by 75.9% of the working parents. Nine remaining people (*n* = 100) indicated a similar answer. Recognition of CF as a chronic disease depended significantly on the professional situation of the parents/guardians (χ^2^_(2)_ = 5355; *p* = 0.069).

Parents/guardians living in cities with over 500,000 inhabitants admitted the least often that their child was hospitalized or had a chronic disease (66.7%). Most often, such statements were made by parents/guardians living in small towns with a population of up to 50,000 (95%) and in large cities with a population of between 150,000 and 500,000 inhabitants (92.9%).

Recognition of CF as a chronic disease by parents/guardians depended significantly on the size of the place of residence of the respondents (χ^2^_(4)_ = 9661; *p* < 0.05) (Table 1).

An analysis comparing the responses of respondents from two professional groups—working parents/guardians and those who resigned from work to care for their child—showed that the latter group assessed the quality of life of their children significantly better in terms of school (M = 78.8) than the working respondents (M = 73.4). The difference between the groups was statistically significant (U = 1586.5; *p* < 0.05) (Table 2).

Parents/guardians with vocational education or lower rated their child’s emotional well-being much higher (M = 85.71) than respondents with secondary education (M = 78.59) and higher (M = 78.43), H = 6.811; *p* < 0.05.

The child’s family relationships were also assessed significantly more highly by people with vocational education or lower (M = 88.81) than by the group of respondents with secondary education (M = 80.55) and higher (M = 79.35), H= 14.1; *p* < 0.01.

The respondents with higher education rated their child’s social interactions significantly lower (M = 75.46) than those with secondary education (M = 80.08) and vocational education or lower (M = 80.48), H = 5.795; *p* = 0.05. The respondents with vocational education or lower assessed the aspect of their child’s coping at school as much better (M = 82.62) than people with secondary education (M = 75.94) and higher (M = 74.26). The difference between the groups was statistically significant (H = 4.923; *p* = 0.085).

Parents/guardians with vocational education or lower rated their children’s quality of life as significantly better (M = 82.95) compared to people with secondary education (M = 77.7) and higher (M = 76.85), H = 7.295; *p* < 0.05) (Table 3).

Correlation analysis showed that the younger the child, the higher his/her quality of life and vice versa. The correlation was negative, low, and statistically significant (rho = −0.279; *p* < 0.01).

The younger the child, the better his/her physical health was assessed. This correlation was negative, very weak, and statistically significant (rho = −0.165; *p* = 0.052).

The younger the children, the higher their scores on the self-esteem scale. This correlation was negative, low, and statistically significant (rho = −0.251; *p* < 0.01). The child’s emotional well-being also correlated negatively with the child’s age. This means that the older the children, the lower their parents assessed their emotional well-being. This correlation was negative, low, and statistically significant (rho = −0.298; *p* < 0.01). In addition, the aspect of school correlated negatively with the age of the child—the older the child, the worse he/she coped with his/her disease, as perceived by the parents. This correlation was negative, low, and statistically significant (rho = −0.354; *p* < 0.01) (Table 4).

The correlation analysis in Table 5 shows that the later the children were diagnosed with cystic fibrosis, the worse their parents assessed their health and vice versa. This correlation was negative, low, and statistically significant (rho = −0.207; *p* < 0.05).

There was a negative, very weak correlation between the age of diagnosis and the child’s emotional well-being (rho = −0.155; *p* = 0.069). This means that the older the child was at the time of diagnosis, the lower the emotional well-being was assessed and vice versa. There was also a very weak, negative correlation between the age of the child diagnosed with CF and his/her self-esteem (rho = −0.168; *p* < 0.05). This means that the older the child was at the time of diagnosis, the worse the parents assess his/her self-esteem and vice versa (Table 5).

## 5. Discussion

Studies on the quality of life of patients constitute an integral part of a comprehensive assessment of the patient’s health. Thanks to them, the therapeutic team can adapt the treatment to accommodate for the patient’s needs as well as safety. In the case of a minor patient, the assessment of the quality of life can be obtained from the child and from the parent/guardian.

To ensure that the family is able to sustain itself as a functional unit, there needs to be someone to look after the entire family. Traditionally, it is the mother who takes care of the sick child. She is the one who takes the child to doctor’s appointments and is responsible for carrying out medical recommendations. In caring for a child with CF, she takes on this heavy burden and is often tired of this responsibility. As she is so tired and busy, she may misunderstand or misinterpret what the doctor is telling her and may not be able to tell her husband or children what they need to know to participate in family activities and care for the child with CF [12], which may have a significant impact on patient safety.

Assessing a child’s well-being becomes particularly important in the case of a chronic disease such as CF. The assessment of health-related quality of life—HRQoL—can be obtained with the use of special questionnaires adapted to the child’s age. They constitute tools measuring the quality of life (QoL) in the pediatric population. The effectiveness of treatment and any side effects that affect daily life may be assessed from the child’s/parent’s point of view. Home therapy versus hospital therapy and drug delivery systems are additional areas where QOL as an outcome measure is valuable. There have been relatively few studies on CF conducted at an adequate level. The measurement of QOL in CF has been fragmentary and largely unreliable [13].

Lin et al. report the KINDL questionnaire as a multidimensional measure of quality of life covering the subdomains of physical, psychological, and social health [14]. Their research has proven that the Parent-Rated Kid-KINDL may be used to measure the quality of life of children with CF. They also pointed to the advantages of the questionnaire that distinguish it from other pediatric QoL tools, e.g., from PedsQL [14]. The Polish version of the questionnaire was used in our research, and it analyzed parents’ opinions on the quality of life of their children [15].

Royce et al., who deal with quality of life in CF, pointed to the significant dependence of the child on the correct application of therapeutic recommendations by their parents, who, thanks to the consistency in treatment, affect not only the physical condition [16] and thus also safety related to the treatment process but also the subjective quality of life. The importance of research on the quality of life was also emphasized by Majkowicz., who saw the correlation between the results of the study and the treatment plan developed by a doctor as well as the possibility of the patient signaling problems related to treatment and its impact on his/her personal life [17]. Problems with treatment are one of the risks related to the occurrence of adverse events.

Researchers dealing with the issue of the quality of life of children and adolescents with CF most often use a tool dedicated to patients with CF—the Cystic Fibrosis Questionnaire and its modified version, the Cystic Fibrosis Questionnaire-Revised (CFQ-R). Schmidt et al. extended their research with a study on the parents of children with CF, proving that questionnaires dedicated to parents are a reliable and valid measure of children’s health-related quality of life. An objective assessment of a child’s health is a component of the overall quality of life of a child affected by a severe genetic disease, i.e., CF. They also point to the correlation between the assessment of the quality of life and the advancement of the disease and its course [18]. In turn, Hegarty et al. showed differences between the perception of the quality of life by the child and the parent, which may suggest a different perception of his/her health by the child, and by the parent/guardian [19]. Blanco-Orive and colleagues conducted a systematic review and verified the availability of tools used to study health-related quality of life (HRQoL) and exercise tolerance in children and adolescents with CF [20].

Havermans et al. analyzed 18 selected scientific studies, 14 of which used the Cystic Fibrosis Questionnaire-Revised (CFQ-R), which has the best characteristics for studying HRQoL in children with CF. These instruments have the best properties for assessing children with CF and are the most reliable, but more research is needed [20].

Our study shows that parents assess the quality of life of children and adolescents with CF at an average level. The study group of 139 parents of children affected by CF who participated in the study assessed their children’s quality of life as good, obtaining an average score (on a 100-point scale examining the quality of life) of 78.17 points. High scores for each HRQoL area were also obtained by Groeneveld et al., who examined a group of Spanish children and their parents [21]. The quality of life of children with CF was similarly assessed by Polish parents in studies using the CFQ-R disease-specific questionnaire [15].

It seems that the occurrence of CF in a child requiring long-term care and parental involvement in treatment is tantamount to the parent’s classification of CF as a chronic disease. Our research shows that not all parents consider CF to be a chronic disease. Our research shows that qualifying CF as a chronic disease does not depend significantly on gender or education, but it does depend significantly on the respondents’ professional situation and where they live. Parents living in large cities (over 500,000 inhabitants) were the least likely to consider their child to have a chronic disease, and such statements were most often made by parents living in small towns (up to 50,000 inhabitants) and in large cities (between 150,000 and 500,000 inhabitants). Our research also shows that the vast majority of parents had resigned from work, and this group classified CF as a chronic disease to the greatest extent. The same results were obtained by Zubrzycka from the Maria Curie-Skłodowska University in Lublin, who, in her research, proved that most parents, mainly mothers, had resigned from professional work to care for a sick child [22]. Jordanian scientists AlAdaileh et al. found that parents of children with CF suffer from difficulties and challenges that limit their daily lives. This may be related to everyday contact with the child, with limitations associated with the disease, being subject to the treatment regimen, and active participation in time-consuming therapies being noticed [23].

Treatment of patients with CF is a long-term, unpredictable process that affects the proper functioning of the family, which is subordinated to restrictive treatment routines for sick children as well as hospitalizations and stays in outpatient clinics. The active participation of at least one of the parents in the treatment of their child is not only related to resignation from professional work but also, as noted by Wong Heriot and, negatively affects the mental state of the caregiver burdened with fear for the future of the child, considering the fact that, despite treatment, the disease is progressive and debilitating, inevitably bringing the child closer to premature death [24]. Such situations pose a threat to the safety of a child suffering from CF from the relatives, e.g., due to fatigue and constant stress.

The results of our research also indicate that it is not only the parents who are concerned about their child’s future. Children are also sad to varying degrees because of their disease, and they are worried that their health may worsen. At the same time, the parents declare that the child is well prepared to cope with the disease.

Although some parents give up work to care for their child and are actively involved in the child’s treatment, their overall assessment of the child’s quality of life is not significantly different from that of a working parent. Our research shows that the quality of life of children and adolescents with CF depends on the parents’ work situation only in relation to school, where the difference between the groups was statistically significant. Parents who had stopped working rated their child’s quality of life in this aspect as significantly better than those who were working.

In the course of our research, it was shown that children relatively often miss something that happens at school. This statement is confirmed by the results of our study, where the vast majority of parents confirmed their children missing classes due to the disease or treatment, which, however, did not significantly affect the child’s fear of his/her future at school. Absence from school is related to the unpredictability of the disease and frequent, sudden infections and bronchopulmonary exacerbations, and may also be the cause of anxiety in parents who are afraid of infection originating in the school setting. This assumption is confirmed by research conducted by Lum et al., who found that students with chronic diseases, including CF, were more likely to have parent-reported learning difficulties and were more likely to have recent disease-related school absences compared to healthy students. These researchers also proved that parents of students with chronic diseases more often reported moderately strong emotional stress in their children and that their children have low social trust compared to parents of healthy students [25]. The results of our research do not support the assumptions of these researchers. According to our results, the children’s social interactions, relationships with peers, and the frequency of meetings with friends were at a high level, and according to the parents, the children did not feel social isolation. What is very important and results from the conducted research is the fact that, according to the opinion of the majority of parents, the children do not feel different from other children and accept their appearance, contrary to the conclusions drawn from the results of a study regarding the different appearance of children with CF, i.e., a slim body structure, the presence of clubbed fingers, or a cough with expectoration of secretions. In addition, the children’s poor appetite and the problem with gaining weight may indicate a different appearance in comparison to peers.

The research results also show a correlation between proper social interactions and parental education. The parents with vocational education or lower assessed the quality of life of their children as significantly better than parents/guardians with secondary education and higher. It was also noticed that, in the opinion of the parents, younger children cope better with the disease than older children. Correlation analysis showed that the younger the child, the higher his/her quality of life and vice versa. This correlation is statistically significant and applies to all health-related quality-of-life areas. Parents of younger children rated their physical well-being, self-esteem, and emotional well-being as better. In addition, the aspect of school correlated negatively with the age of the child—the older the child, the worse he/she copes in this area in the opinion of the parents. This is confirmed by a study conducted by researchers from the Jagiellonian University in Poland (Tobiasz-Adamczyk. et al.) on the parents of children suffering from CF, who showed that the quality of life of people suffering from CF was assessed lower in many dimensions in older age groups [26]. This confirms the correlation between the age of the child and the advancement of the disease. The age at which CF was diagnosed also affects the child’s quality of life.

Our research confirmed the assumption that the quality of life in the emotional dimension depends on the age of the child diagnosed with CF. The analysis of the results show that the later CF was diagnosed in children, the worse their parents assessed their health and vice versa. This applies mainly to the researched sphere of self-esteem and emotional well-being. Not all children were covered by the screening program, which detects CF. This program has been operating throughout Poland since July 2009.

Studies have shown that some children who were born before 2009 were not tested for CF. However, in some children who had a newborn screening test, the result did not confirm CF.

The analysis of the results also showed that the vast majority of parents found out about the disease in the first month of their child’s life and some later, sometimes at an advanced age. Coffey et al. also confirmed the differences in the results between early and late detection of CF. In the era of neonatal examinations, they also pointed to worse health status before diagnosis and worse results of vital parameters after diagnosis [27]. These results may suggest better preparation of parents to care for a sick child when the diagnosis was made early and thus better well-being of the child and better physical performance. Early diagnosis and comprehensive treatment significantly improve the quality of life of the child, which is confirmed by the results of research published in Klinische Pediatrie by Schütz et al. drawing attention to the importance of screening tests to improve the course of CF in children [28].

A review of publications on the quality of life of children and adolescents with CF shows how the development of medicine and improving the quality of comprehensive care extends the life of these patients and improves their subjective quality of life, because the increase in objective quality is reflected in the results of physical examinations. The past six decades have brought remarkable improvements in health outcomes for people with CF, which was once a fatal disease for infants and young children. Although the life expectancy of people with CF has increased significantly, the disease continues to limit survival and quality of life and is a heavy burden for patients and their families. Members of the Lancet Respiratory Medicine Committee on the Future of Cystic Fibrosis Care (Bell et al.) pointed to various challenges related to the changing landscape of cystic fibrosis care and the possibilities for progress in its treatment, with particular emphasis on advances in CFTR-modulating therapies to address the underlying CF defect [29]. The introduction of causal treatment on a global scale is associated with the hope that in a few years CF will cease to be a fatal disease and will be classified as a chronic disease. Causal treatment will also be a good indicator for undertaking further studies comparing the quality of life in each age group of patients with CF.

The results show that qualifying CF as a chronic disease depends significantly on the place of residence of the parents, does not depend on the gender and education of the parents, and does depend on their professional situation. In turn, the quality of life of children and adolescents with CF significantly depends on the professional situation of their parents only in the aspect of school. Proper social interactions depend on the level of parental education. The quality of life of children and adolescents with CF depends on age—the younger the child, the higher his/her quality of life and vice versa. This correlation applies to aspects of the quality of life related to physical health, emotional well-being of the child, and school. The quality of life in the emotional dimension depends on the age of the child at diagnosis.

## 6. Conclusions

The Kid- & Kiddo-KINDLR QoL Questionnaire emerged as a valuable instrument for assessing parental awareness in children with CF. The research highlights the necessity for the entire family to comprehend and acknowledge the profound influence of CF on family dynamics. Fatigue or misconceptions among parents might lead to misinterpretations of medical instructions, thereby impacting both family life and patient safety. To ensure the utmost safety of the patient, parents should collaborate closely with healthcare professionals not only during hospital or clinic visits but also within the home environment. It is essential to consider the well-being of the entire family, addressing the needs of all members rather than solely focusing on the challenges faced by the child with CF.

### Inclusion Criterion

The research was conducted in the Department of Lung Diseases—Cystic Fibrosis Treatment Center of the Independent Complex of Public Health Care Institutions, named after Children of Warsaw in Dziekanów Leśny in Łomianki in the period from 6 July to 1 December 2021. The study was conducted after obtaining the consent of the Hospital Management and the Head of the Department of Pulmonary Diseases—Cystic Fibrosis Treatment Center of the above-mentioned hospital.

Study participants were recruited from parents who met the following criteria:

(a) Diagnosis of CF in the child;
(b) Age of the child 7–17 years (the Kid- & Kiddo-KINDLR questionnaire was used—the version intended for parents of children aged 7–17 years);
(c) The child’s place of residence after discharge from the hospital is home.

Parents were informed about how to complete the questionnaires after prior consent to the survey, and there was full anonymity with the survey along with compliance with the principles of the GDPR [30,31].

The second questionnaire was authors’ questionnaire. The demographic part contained information about the parents, such as education level, gender, age, occupation, and education. The remaining questions concerned, among others, CF diagnostics and daily respiratory rehabilitation.

A control group was not taken into account. The study included a group of parents of children with CF. It was a representative group of the entire population of children with CF in Poland.

## Figures and Tables

**Table 1:** Recognition of CF as a chronic disease by parents/guardians and the place of residence of the respondents.

Recognition of CF as a Chronic Disease by Parents/Guardians		Place of Residence				
		Countryside	Town up to 50,000	City from 50,000 to 150,000	City from 150,000 to 500,000	Above 500,000
Yes	*n*	37	38	15	13	14
	%	82.2%	95.0%	78.9%	92.9%	66.7%
No	*n*	8	2	4	1	7
	%	17.8%	5.0%	21.1%	7.1%	33.3%

**Table 2:** Descriptive statistics of individual scales of the KINDL questionnaire by professional status.

Descriptive Statistics of Individual Scales of the KINDL Questionnaire	Professional Status					
	Working (*n* = 54)		Resigned from Work to Take Care of the Child (*n* = 76)			
	M	SD	M	SD	U	*p*
Physical health	78.9	14.56	78.5	15.60	2011.0	0.845
Emotional well-being	78.1	13.65	80.2	13.48	1808.5	0.246
Self-esteem	74.4	13.20	73.9	16.01	1981.0	0.735
Family	80.0	10.37	82.2	10.69	1769.5	0.177
Social contacts	78.4	12.09	78.2	16.37	1959.0	0.657
School	73.4	14.60	78.8	14.19	1586.5	0.027
Disease	69.9	13.93	70.3	16.15	1380.0	0.866
Quality of life of children	77.4	9.76	78.7	10.88	1858.0	0.359

**Table 3:** Descriptive statistics of individual scales of the KINDL questionnaire, broken down by education.

Descriptive Statistics of Individual Scales of the KINDL Questionnaire	Education							
	Vocational or Lower (*n* = 21)		Secondary (*n* = 64)		Higher (*n* = 54)		H	*p*
	M	SD	M	SD	M	SD		
Physical health	83.57	14.76	76.88	14.60	78.98	14.55	3.243	0.198
Emotional well-being	85.71	14.86	78.59	13.41	78.43	11.93	6.811	0.033
Self-esteem	76.43	16.21	73.44	15.25	74.17	13.09	0.867	0.648
Family	88.81	9.34	80.55	10.08	79.35	10.73	14.100	0.001
Social contacts	80.48	17.67	80.08	14.49	75.46	13.75	5.974	0.050
School	82.62	12.21	75.94	13.80	74.26	15.46	4.923	0.085
Disease	75.05	12.79	70.34	16.08	67.76	14.03	3.067	0.216
Quality of life of children	82.95	11.03	77.70	10.10	76.85	9.79	7.295	0.026

**Table 4:** Spearman’s rho correlations between child’s age and quality of life.

Quality of Life		Age
Physical health	rho	−0.165
	*p*	0.052
Emotional well-being	rho	−0.298
	*p*	0.000
Self-esteem	rho	−0.251
	*p*	0.003
Family	rho	−0.085
	*p*	0.319
Social contacts	rho	−0.078
	*p*	0.360
School	rho	−0.354
	*p*	0.000
Disease	rho	−0.134
	*p*	0.147
Quality of life of children	rho	−0.279
	*p*	0.001

**Table 5:** Spearman’s rho correlations between the age of the child at CF diagnosis and the child’s quality of life.

		Age of the Child at CF Diagnosis
Physical well-being	rho	−0.207
	*p*	0.014
Emotional well-being	rho	−0.155
	*p*	0.069
Self-esteem	rho	−0.168
	*p*	0.047
Family	rho	0.056
	*p*	0.515
Social contacts	rho	0.036
	*p*	0.675
School	rho	−0.143
	*p*	0.094
Disease	rho	−0.171
	*p*	0.064
Quality of life of children	rho	−0.130
	*p*	0.126

## Data Availability

Not applicable.
